# P-1717. Temporal-spatial contributions of Candida albicans environmental response genes to pathogenesis of intra-abdominal candidiasis (IAC)

**DOI:** 10.1093/ofid/ofaf695.1888

**Published:** 2026-01-11

**Authors:** Shaoji Cheng, M Hong Nguyen, Cornelius J Clancy

**Affiliations:** University of Pittsburgh, Pittsburgh, Pennsylvania; University of Pittsburgh Medical Center, Pittsburgh, Pennsylvania; University of Pittsburgh, Pittsburgh, Pennsylvania

## Abstract

**Background:**

IAC, which includes peritonitis and intra-abdominal abscesses (IAAs), is the second most common type of invasive candidiasis, but its pathogenesis is poorly understood.

**Table 1.**
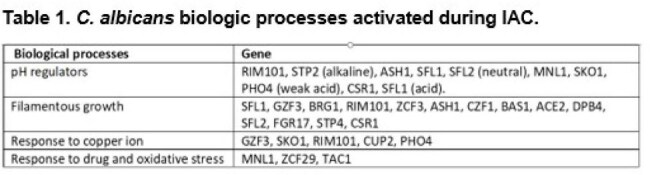
C. albicans biologic processes activated during IAC.

**Figure 1.**
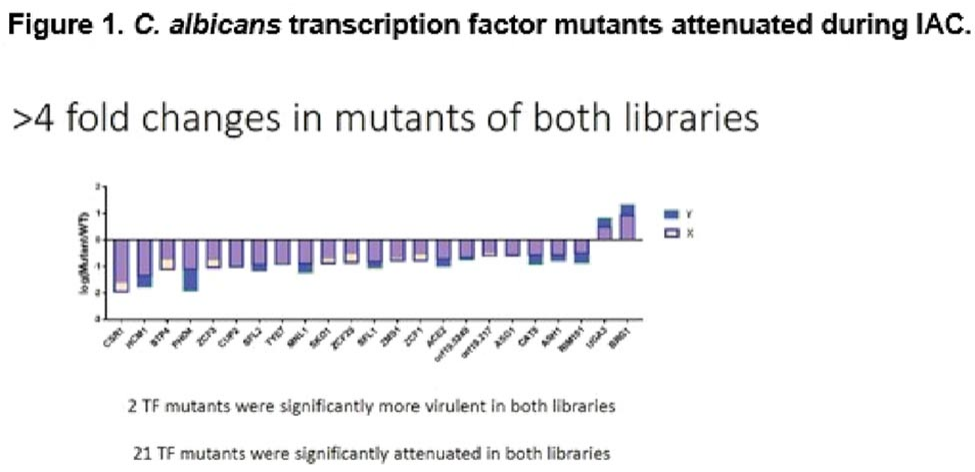
C. ablicans transcription factor mutants attenuated during IAC.

**Methods:**

We used RNA-Seq to measure C. albicans SC5314 gene expression during early peritonitis (30-min), late peritonitis (24-hr) IAA (48-hr) of mice. ≥2-fold differences were significant (false discovery ≤0.01). We screened duplicate signature-tagged, homozygous deletion libraries for 165 C. albicans transcription factors (TFs) in 72-hr IAA.

**Figure 2.**
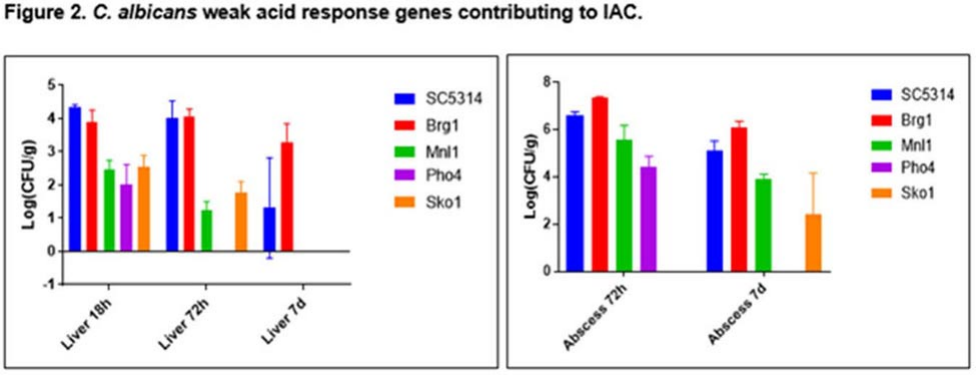
C. albicans weak acid response genes contributing to IAC.

**Figure 3.**
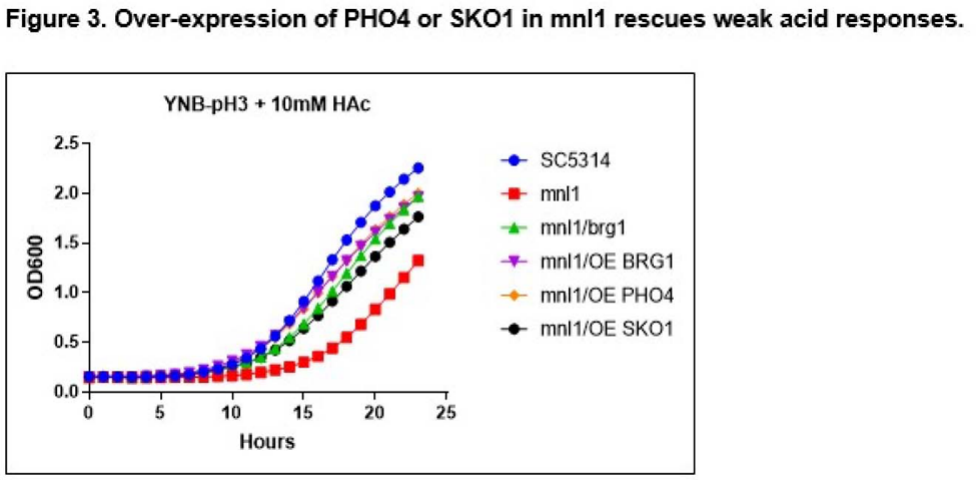
Over-expression of PHO4 or SKO1 in mnl1 rescues weak acid responses.

**Results:**

The 50 genes most highly expressed during early peritonitis were associated with pH (e.g., RIM101, PHR1), oxidative stress responses (e.g., SOD4-6), and adhesion/hyphal growth (e.g., ALS3, HWP1, ECM331, SAP6). The corresponding 50 late peritonitis genes were associated with phagocyte responses and nutrient acquisition (glyoxylate cycle, fatty acid β-oxidation, iron homeostasis). Responses within IAA included DNA damage and iron metabolism. Null mutants for genes involved in adhesion (ALS1, ALS3), transport (OPT8, SGE11), biofilm (ZCF23), DNA damage responses (RFX1, RFX2, DDI1), cell wall responses (DAP2) and copper metabolism (CCC2) were attenuated during peritonitis and/or IAA [Table 1]. 21 TF mutants were significantly attenuated for virulence in both libraries [Fig 1]. Biologic processes over-represented were regulation of pH responses, biofilm, hyphal formation, echinocandin responses, and copper metabolism. 9 pH response regulators were confirmed to contributed to virulence, including RIM101, STP2 (alkaline), ASH1, SFL1, SFL2 (neutral), MNL1, SKO1, PHO4 (weak acid), and CSR1 (acid) [Fig 2]. Over-expression of aspartyl protease SAP5 in rim101 restored virulence during IAA at 3, 7, and 10 days. Over-expression of either SKO1 or PHO4 in mnl1 restored weak acid responses in vitro [Fig 3] and virulence (not shown).

**Conclusion:**

Numerous C. albicans environmental response genes make temporal-spatial contributions to IAC. pH response regulators RIM101 and MNL1 contribute to virulence during peritonitis and IAA, in part by regulating SAP5 and PHO4/SKO1, respectively. Other processes and genes involved in IAC pathogeneses are adhesion, transport, biofilm, DNA damage responses, cell wall responses and copper metabolism.

**Disclosures:**

M Hong Nguyen, MD, Basilea: Advisor/Consultant|BioMerieux: Grant/Research Support|Melinta: Grant/Research Support|Pulmocide: Advisor/Consultant|Pulmocide: Grant/Research Support Cornelius J. Clancy, MD, Merck: Grant/Research Support|Shionogi: Advisor/Consultant

